# Development and Validation of a Deep Learning System for the Detection of Nondisplaced Femoral Neck Fractures

**DOI:** 10.3390/bioengineering12050466

**Published:** 2025-04-28

**Authors:** Lianxin Wang, Ce Zhang, Yaozong Wang, Xin Yue, Yunbang Liang, Naikun Sun

**Affiliations:** 1 Department of Orthopedics, The First Affiliated Hospital of Xiamen University, School of Medicine, Xiamen University, Xiamen 361003, China; dr_shepherd@sina.com; 2 Department of Anesthesiology, Xiang’an Hospital of Xiamen University, School of Medicine, Xiamen University, Xiamen 361101, China; zhangcemelody88@163.com; 3 Department of Orthopedics, Zhongshan Hospital Xiamen University, School of Medicine, Xiamen University, Xiamen 361003, China; xmzswyz@xmu.edu.cn; 4 Department of Radiology, Zhongshan Hospital Xiamen University, School of Medicine, Xiamen University, Xiamen 361004, China; yuexin7504@163.com

**Keywords:** deep learning, femoral neck fracture, convolutional neural network

## Abstract

Hip fractures pose a significant challenge to healthcare systems due to their high costs and associated mortality rates, with femoral neck fractures accounting for nearly half of all hip fractures. This study addresses the challenge of diagnosing nondisplaced femoral neck fractures, which are often difficult to detect with standard radiographs, especially in elderly patients. This research evaluates a deep learning model that employs a convolutional neural network (CNN) within a ResNet framework, designed to enhance diagnostic accuracy for nondisplaced femoral neck fractures. The model was trained and validated on a dataset of 2032 hip radiographs from two hospitals, with additional external validation performed on datasets from other institutions. The AI model achieved an accuracy of 94.8% and an Area Under Curve of 0.991 on anteroposterior pelvic/hip radiographs, outperforming emergency physicians and delivering results comparable to expert physicians. External validation confirmed the model’s robust accuracy and generalizability across diverse datasets. This study underscores the potential of deep learning models to act as a supplementary tool in clinical settings, potentially reducing diagnostic errors and improving patient outcomes by facilitating a quicker diagnosis and treatment.

## 1. Introduction

Hip fractures are a significant issue in the elderly population, accounting for more than 14% of all fractures [1]. However, they represent a substantial 72% of fracture-related healthcare costs [2]. In the US alone, there are approximately 300,000 hip fractures each year [3,4], and this number is expected to increase due to an aging population [3,5]. By 2050, it is projected that there will be 6.3 million hip fractures worldwide, costing around USD 131.5 billion annually [6,7]. Hip fractures, especially in older patients, have a profound impact on functional status and carry a high risk of mortality, with reported 1-year mortality rates as high as 30% [8,9].

**Figure 1:**
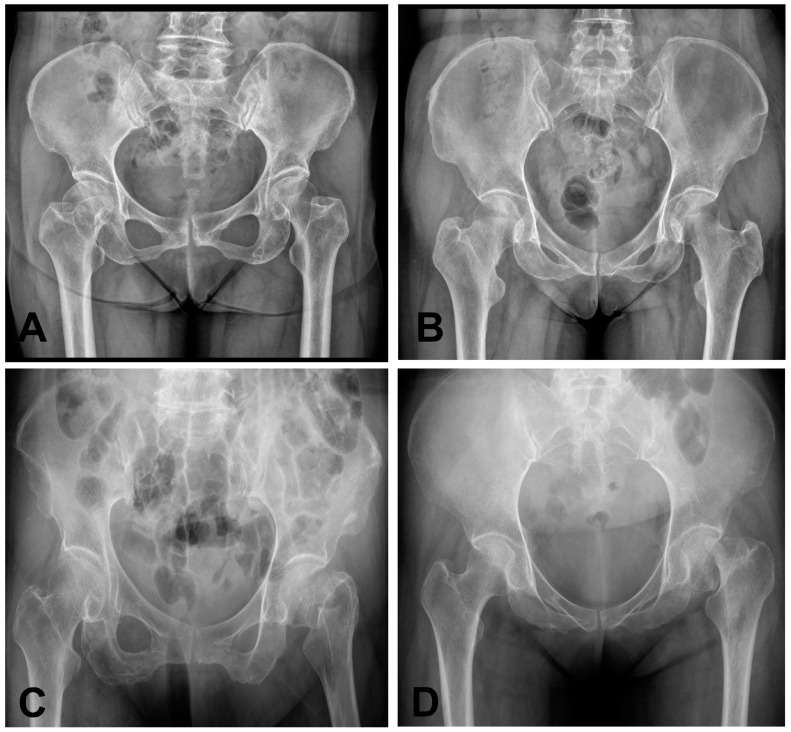
Representative radiographs of different femoral neck fracture cases. (**A**,**B**) Shows radiographs of nondisplaced femoral neck fractures (Garden I/II), while (**C**,**D**) are displaced femoral neck fractures (Garden III/IV).

While plain X-rays are commonly used as an initial screening test for hip fractures, up to 10% of these fractures can be occult and not visible on radiographs [10]. While diagnosing displaced femoral neck fractures and intertrochanteric fractures is usually straightforward, detecting nondisplaced femoral neck fractures is challenging due to their subtle appearance (Figure 1) [11,12,13].

In such cases, further imaging techniques like a CT, bone scan, and MRI are often required for diagnosis. This not only increases diagnostic costs, but also places a burden on doctors and patients, as well as resource utilization. Moreover, occult fractures can lead to delayed diagnoses and worse patient outcomes, including increased mortality rates [14,15], longer hospital stays [16], and higher costs of care [17].

Improving the efficiency of hip fracture diagnoses at the first clinical presentation could potentially reduce both the harm and costs. Deep learning, particularly artificial neural networks, have shown promise in achieving human or near-human-level performance in complex perceptual tasks, such as image classification and natural language processing. One specific deep learning construct commonly used for image recognition tasks is a convolutional neural network (CNN), which allows a system to automatically extract features relevant to a specific problem without explicit human instruction. However, the sensitivity of CNNs in identifying nondisplaced femoral neck fractures is relatively low [18,19], and the performance of the reported models has shown a significant decrease during external validation [20,21].

In this study, we aim to evaluate the performance of a deep learning model in detecting nondisplaced femoral neck fractures. We compared the model’s performance with that of various physicians, including orthopedic surgeons, radiologists, and emergency physicians. Additionally, we conducted external validation to assess the robustness and generalizability of this CNN technique.

## 2. Materials and Methods

This study was conducted at two tertiary hospitals between May 2023 and July 2023, utilizing anteroposterior (AP) pelvic/hip radiographs from June 2009 to May 2023. Approval for this study was obtained from the Institutional Review Boards (IRBs) of The First Affiliated Hospital of Xiamen University (No. 2022063) and Zhongshan Hospital of Xiamen University (No. 2023079). The IRBs of both hospitals waived the requirement for informed consent. All methods and procedures adhered to the principles outlined in the Declaration of Helsinki.

### 2.1. DataSet Acquisition

**Figure 2:**
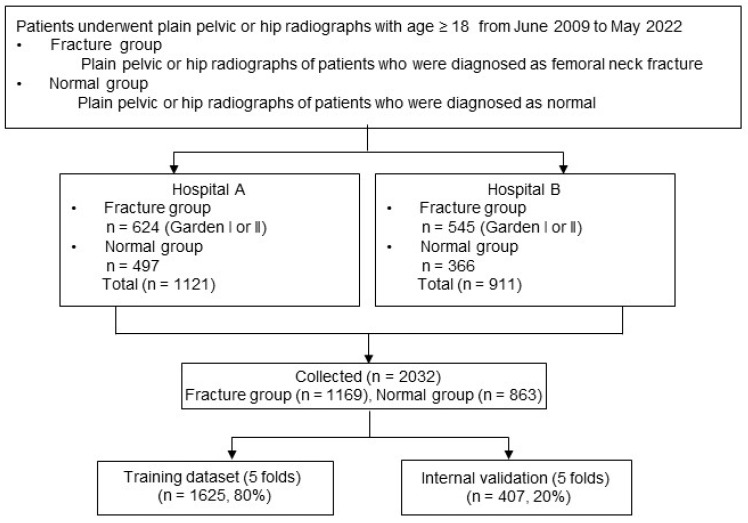
Flowchart of patient enrollment.

A search was conducted in the radiology reports of The First Affiliated Hospital of Xiamen University and Zhongshan Hospital of Xiamen University, covering the period from June 2009 to May 2023. The flowchart of patient enrollment is shown in Figure 2.

A total of 4142 cases of femoral neck fractures in patients aged 18 years or older were initially identified. Hip radiographs were then extracted as Digital Imaging and Communications in Medicine (DICOM) files from the picture archiving and communication system (PACS). These cases were reviewed by a group of orthopedic surgeons, who confirmed 1169 cases as nondisplaced femoral neck fractures (Garden I/II) after excluding 2973 cases of displaced fractures (Garden III/IV).

The ground truth for fracture status was determined through a consensus among three orthopedic surgeons (LX Wang, YZ Wang, and NK Sun), taking into account CT scans, MRI scans, and postoperative radiographs. To establish a comparison group, normal hip radiographs were obtained from patients diagnosed as normal in the reports by two board-certified radiologists. Orthopedic surgeons then reviewed these radiographs to ensure the absence of any fractures. To prevent an overfitting of the Convolutional Neural Network (CNN), only one hip image per patient was used from the normal hip radiographs, except for the cases of non-fracture AP radiographs. In total, the dataset comprised 2032 AP hip radiographs, consisting of 863 normal hips and 1169 hip radiographs with fractures.

### 2.2. Image Preprocessing

**Figure 3:**
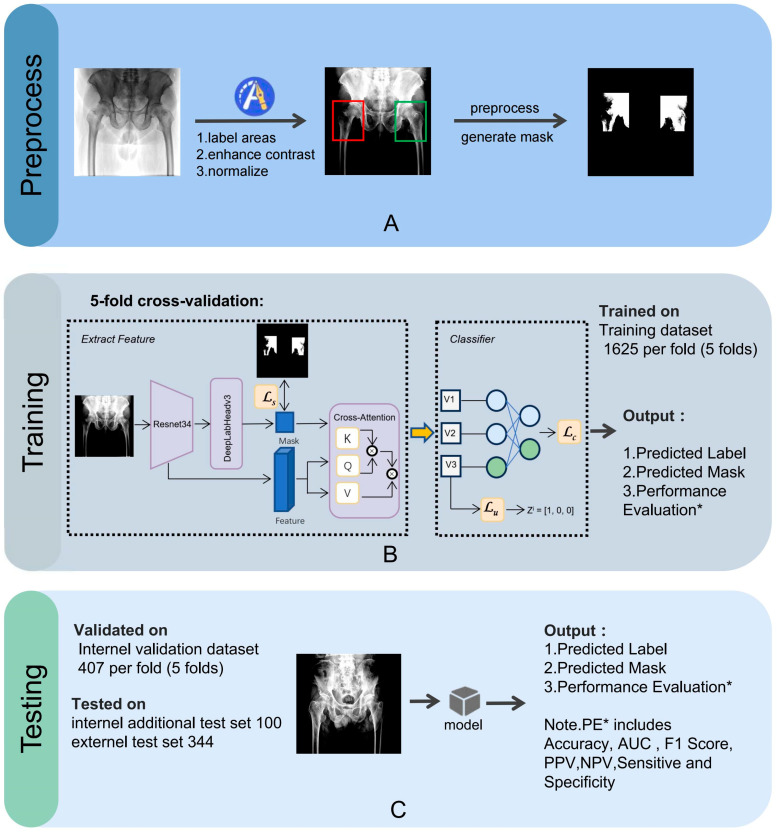
The preprocessing steps (labeling areas, enhancing contrast, normalizing) (**A**) and the model’s training (**B**) and validation processes (**C**), using 5-fold cross-validation on the training dataset (1625 samples per fold) and internal validation dataset (407 samples per fold), with additional testing on internal (100 samples) and external (344 samples) test sets. Performance evaluations include sensitivity, specificity, accuracy, PPV, NPV, F1 score, and AUC. AUC, area under the curve; PPV, positive predicted value; NPV, negative predicted value.

The image preprocessing, training, and testing processes for the CNN model are illustrated in Figure 3.

An orthopedic surgeon, LX Wang, drew bounding boxes around each femoral neck, including the femoral head and the greater and lesser trochanters in the AP hip radiographs. We then applied normalization and contrast enhancement to generate three-channel grayscale images for model training. A mask binary image was generated based on the bounding box. We performed five-fold cross-validation on the entire dataset, a widely employed approach to rigorously evaluate the model’s performance, ensuring its consistency and dependability. All images were randomly split into a training set (80%) and a testing set (20%).

### 2.3. Model Architecture

**Figure 4:**
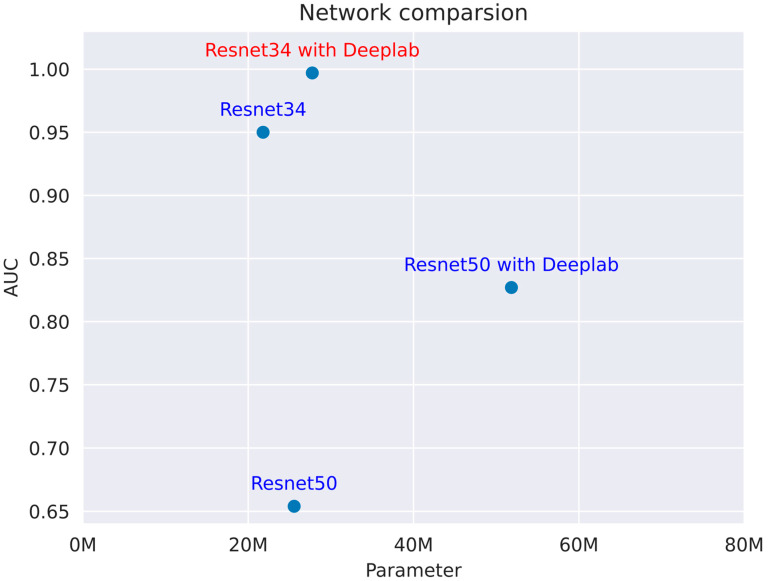
This figure compares the performance of the Resnet34 and Resnet50 models, both with and without the Deeplab extension, across a parameter range of 0 M to 80 M, with performance metrics ranging from 1.00 to 0.65.

Unlike the conventional ResNet classification model, we have developed and validated a novel deep learning image classification algorithm within the ResNet framework. The network comparison, shown in Figure 4, presents the performance of the Resnet34 and Resnet50 models, both with and without the Deeplab extension. Our experiments show ResNet34–DeepLab achieved superior fracture detection sensitivity because its shallower architecture better preserves subtle fracture features (<0.5 mm) while its 512 px receptive field optimally matches typical femoral neck fracture dimensions (450–550 px).

Performance metrics, indicated by numerical values ranging from 1.00 to 0.65, are plotted against the number of parameters (in millions) on the *x*-axis. The data suggest that the Resnet34 model with Deeplab outperforms both the standard Resnet34 and Resnet50 models, as well as the Resnet50 model with Deeplab, across the parameter range of 0 M to 80 M, highlighting the efficiency and effectiveness of the Resnet34 with Deeplab configuration in handling the given tasks.

This approach enables the classification network to focus on relevant regions and capture more information. Since our model receives complete X-ray images, which may contain various non-fracture bone joint interferences, we guide the model’s attention toward the fracture area based on key semantic regions. Specifically, our model first segments the location of the femoral neck and then leverages this semantic information to direct its attention to the femoral neck region in the original image. Subsequently, we employ a concept encoder to translate the feature maps generated by the convolutional neural network into human-interpretable concepts. Each concept is represented by a filter, and we enforce the activation of these filters when the corresponding concepts exist in the original image, which is achieved through weighted cross-entropy loss.

The prediction results are obtained by feeding the high-level features extracted from the previous two stages into a fully connected network. During the training process, the model undergoes 100 epochs and updates the parameters using batch gradient algorithms with a batch size of 16. The final pipeline’s accuracy can be fine-tuned by adjusting the score threshold returned by the softmax algorithm, providing precise results for different X-ray images.

### 2.4. Model Evaluation

The performance of our model was evaluated and the probability of a fracture was determined. The final classification (fracture/no fracture) was based on a probability cut-off value of 0.5. To visualize important regions in the image for diagnosis, we employed Grad-CAM [22], as our proposed network consists of CNNs with fully connected layers. Consequently, Grad-CAM was applied to the last convolutional layer before the fully connected layer to verify medical diagnoses.

We compared the performance of our model to that of ten clinical physicians using 100 images from an independent dataset. This dataset consisted of anteroposterior (AP) hip/pelvic radiographs, including 50 normal cases and 50 cases with fractures. The expert group included three orthopedic surgeons with 16 to 30 years of experience and two radiologists with 4 and 10 years of experience, respectively. Additionally, there were five emergency physicians with 5 to 9 years of experience. Each physician reviewed the images, which were presented in the same quality as those input into the model, and classified each image as either “fracture” or “no fracture”.

Additionally, we utilized an external dataset to assess the performance of our model. The dataset, obtained from The Second Hospital of Jilin University, consisted of 344 AP hip radiographs, including 177 normal hips and 167 hips with fractures. All images were diagnosed as nondisplaced femoral neck fractures or normal by a consensus of four experienced orthopedic surgeons (LX Wang, YZ Wang, Weibo Jiang, and Naikun Sun).

To evaluate the performance of our model, we calculated several metrics, including sensitivity, specificity, accuracy, positive predicted value (PPV) and negative predicted value (NPV), F1 score, receiver operating characteristic (ROC), and area under the curve (AUC). All statistical analyses were performed using the extension packages “scikit-learn,” “scipy,” and “pandas”. The pipeline of the model was built on an Ubuntu 18.04 operating system with the PyTorch 1.12.1+cu113 open-source library, using Python 3.9.0 (Python Software Foundation).

## 3. Results

The patient information from the datasets is summarized in Table 1.

The development dataset comprises 2032 patients with an average age of 64.6 years (±17.5 years). Among these, 1270 patients (62.5%) are female, and 762 patients (37.5%) are male. Within this dataset, 863 patients (42.5%) have no fractures, while 1169 patients (57.5%) have fractures. The independent test dataset includes 100 patients with a higher average age of 73.5 years (±14.4 years). Of these, 65 patients (65.0%) are female, and 35 patients (35.0%) are male. The dataset is evenly split with 50 patients (50.0%) having no fractures and 50 patients (50.0%) having fractures. The external validation dataset consists of 344 patients with an average age of 60.0 years (±17.9 years). This dataset has 202 female patients (58.7%) and 142 male patients (41.3%). In terms of fracture status, 177 patients (51.5%) have no fractures, and 167 patients (48.5%) have fractures. These datasets provide a comprehensive overview of patient demographics and fracture statuses, facilitating the development, testing, and validation of the deep learning model.

### 3.1. Performance of the AI Model

The model’s diagnostic performance is impressive, as shown in Table 2.

**Figure 5:**
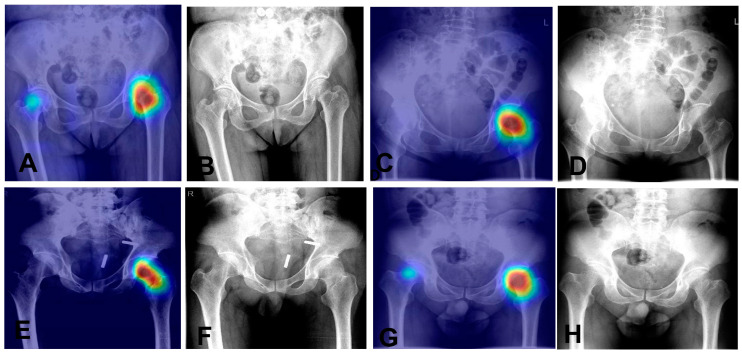
Heatmaps and corresponding X-ray images of four nondisplaced femoral neck fracture cases. (**A**,**B**): Heatmap (**A**) and original X-ray (**B**) of Case 1. (**C**,**D**) Heatmap (**C**) and original X-ray (**D**) of Case 2. (**E**,**F**): Heatmap (**E**) and original X-ray (**F**) of Case 3. (**G**,**H**): Heatmap (**G**) and original X-ray (**H**) of Case 4.

It has a sensitivity of 97.5% and a specificity of 95.1%, indicating it effectively recognizes both positive and negative cases. Overall, the model achieves an accuracy of 96.5%. The positive predictive value (PPV) is 96.5%, and the negative predictive value (NPV) is 96.6%, reflecting its reliability in predicting outcomes. The F1 score is 97.0%, highlighting the model’s balanced precision and recall. These results demonstrate the model’s high accuracy and reliability in diagnostics. We also utilized Grad—CAM maps for further analysis of our model’s behavior (as shown in Figure 5). The model is capable of accurately localizing the region of the nondisplaced femoral neck fracture.

### 3.2. Comparison with Clinical Physicians in Independent Datasets

The diagnostic performance of the AI model compared to 10 physicians, including both experts and emergency physicians, is highlighted in Table 3.

The AI model demonstrates a sensitivity of 96.0%, a specificity of 93.6%, and an overall accuracy of 94.8%. Its positive predictive value (PPV) is 93.8%, and the negative predictive value (NPV) is 95.9%, with an F1 score of 94.9%. In comparison, the expert physicians have a lower sensitivity of 88.8%. Their overall accuracy is 92.6%, with a PPV of 96.3% and an NPV of 89.7%, resulting in an F1 score of 92.3%. The differences in sensitivity and NPV between the AI and the experts are statistically significant (*p* = 0.005 and *p* = 0.004, respectively).

The emergency physicians perform with a sensitivity of 71.2% and a specificity of 95.6%. Their overall accuracy is 83.4%, with a PPV of 94.4% and an NPV of 77.0%, and an F1 score of 81.0%. The differences in sensitivity, accuracy, NPV, and F1 score between the AI and the emergency physicians are statistically significant (*p* < 0.05). These comparisons underscore the AI model’s superior diagnostic performance, particularly in sensitivity and overall accuracy, when compared to both expert and emergency physicians.

**Figure 6:**
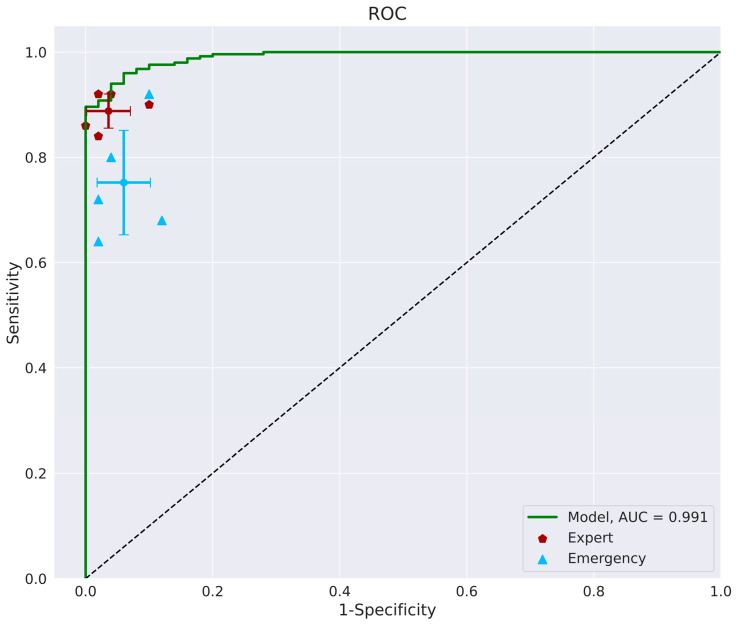
The ROC curve of the model versus that of the experts (orthopedic surgeons and radiologists, in red) and emergency physicians (in blue). The AUC of the model was 0.991. ROC, receiver operating characteristic; AUC, area under the curve.

The model achieved an impressive AUC of 0.991 (Figure 6), indicating a high level of accuracy.

**Figure 7:**
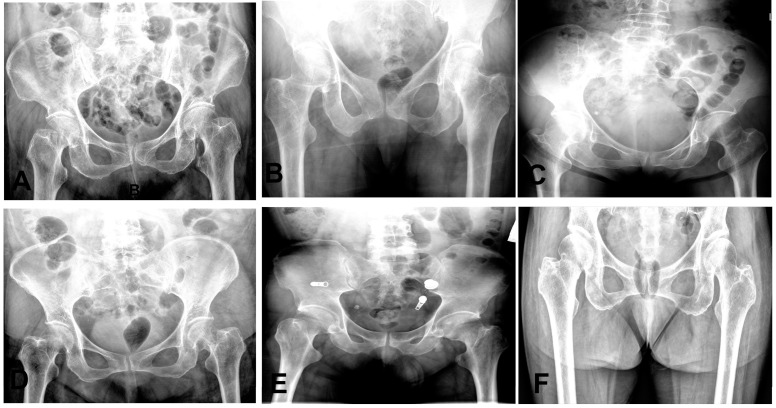
Representative radiographs of six nondisplaced femoral neck fractures initially diagnosed as normal on anteroposterior hip X-rays. (**A**,**F**): Right hip fractures (two cases). (**B**–**E**): Left hip fractures (four cases).

The 95% confidence intervals for both the mean sensitivity and mean specificity of the emergency group were below the ROC curve of the model. We also observed that some cases of fractures were diagnosed as normal by some clinicians, as shown in Figure 7. These results highlight the potential value of our AI model in improving diagnostic accuracy and reducing the risk of misdiagnosis for femoral neck fractures.

### 3.3. Performance of the AI Model in External Validation

The AI model showed strong diagnostic performance on the external dataset from The Second Hospital of Jilin University (Table 4).

The model achieved a sensitivity of 93.9% and a specificity of 94.2%. Its overall accuracy was 94.0%, with a positive predictive value (PPV) of 93.9% and a negative predictive value (NPV) of 94.3%. The F1 score was 93.8%. These results demonstrate that our AI model has good generalizability and can perform well on different datasets, suggesting its potential for use in different clinical institutions.

## 4. Discussion

Our study highlighted the capability of a CNN model to accurately distinguish nondisplaced femoral neck fractures from normal hips using anteroposterior pelvic/hip radiographs. The model achieved an impressive accuracy of 94.8% and an AUC of 0.991, surpassing the performance of the emergency physicians. These high metrics underscore the model’s potential to enhance diagnostic precision across various test datasets.

Diagnosing nondisplaced femoral neck fractures can be challenging, especially for nonexpert physicians, as our findings also show. While previous studies have explored CNNs for this purpose, their results were less effective. For instance, Krogue reported a CNN model with a sensitivity of just 51.2% using 182 cases, and Mutasa achieved a sensitivity of 54% with 127 cases. These limited outcomes were likely influenced by the small size of their datasets.

Our study emphasizes the advantages of using a larger sample size to train our CNN model, which significantly contributed to its superior performance in diagnosing nondisplaced femoral neck fractures. Unlike previous studies, such as those by Justin D and Simukayi, which were limited by smaller datasets and achieved sensitivities of only 51.2% and 54%, respectively, our model achieved a remarkable accuracy of 94.8% and an AUC of 0.991. This highlights the robustness and reliability of our approach, demonstrating its potential to greatly improve diagnostic accuracy over traditional methods and models trained on smaller datasets. The extensive dataset used in our study allowed the model to learn more effectively, capturing subtle differences in anteroposterior pelvic/hip radiographs that nonexpert physicians might miss, thereby enhancing its diagnostic capability.

Our model could serve as a valuable second reader in clinical environments, helping to reduce misdiagnoses and deliver expert-level results for nondisplaced femoral neck fractures. By detecting fractures in real time, it can minimize the need for additional examinations and shorten the time to surgery, which may lead to better patient outcomes. Furthermore, the model can act as a supportive tool to boost the diagnostic performance of physicians, including the radiologists responsible for finalizing radiograph reports.

It is important to recognize the limitations of our study. First, the retrospective case-control design might introduce potential biases, particularly in overestimating diagnostic accuracy as it compared definitive fracture cases with clearly healthy controls. To achieve a more comprehensive understanding, larger prospective studies with diverse participant groups are necessary to evaluate the model’s performance and verify its generalizability. Importantly, the predictive values (PPV/NPV) reported here should be interpreted with caution due to the artificially controlled prevalence in our study design. Second, a fracture diagnosis relies not only on radiological features but also on the patient’s medical history and clinical presentation. Our study focused solely on radiological aspects, which may have restricted the accuracy for clinical physicians. Additionally, we did not perform age-specific comparisons, even though femoral neck fractures present and are managed differently across various age groups. Moreover, the manual marking of regions of interest (ROI) is labor-intensive and not practical for clinical applications.

In summary, our study highlights the potential of our CNN model to enhance the accuracy of diagnosing nondisplaced femoral neck fractures using anteroposterior pelvic/hip radiographs. The model shows promise in reducing diagnostic errors and expediting the process of diagnosis and surgery, potentially leading to improved patient recovery and reduced morbidity. Additional research is required to confirm the generalizability of our results and to address the study’s limitations.

## Figures and Tables

**Table 1:** Datasets characteristics.

Parameter	Development Dataset (*n* = 2032)	Independent Test Dataset (*n* = 100)	External Validation Dataset (*n* = 344)
Age, years	64.6 ± 17.5	73.5 ± 14.4	60.0 ± 17.9
Sex			
Female	1270 (62.5%)	65 (65.0%)	202 (58.7%)
Male	762 (37.5%)	35 (35.0%)	142 (41.3%)
No fracture	863 (42.5%)	50 (50.0%)	177 (51.5%)
Fracture	1169 (57.5%)	50 (50.0%)	167 (48.5%)

Data are means ± standard deviations, and the number of patients/images and the associated population percentages are in parentheses.

**Table 2:** Diagnostic performance of the model.

Parameter	Sensitivity (%)	Specificity (%)	Accuracy (%)	PPV (%)	NPV (%)	F1_Score (%)
Model	97.5 (96.2, 98.8)	95.1 (92.9, 97.3)	96.5 (95.7, 97.3)	96.5 (95.0, 98.0)	96.6 (94.8, 98.4)	97.0 (96.3, 97.7)

Numbers in parentheses are 95% CIs. Cl, confidence interval.

**Table 3:** Diagnostic performance of AI and 10 physicians.

AI/physicians	Sensitivity (%)	Specificity (%)	Accuracy (%)	PPV (%)	NPV (%)	F1_Score (%)
AI	96.0 (94.2, 97.8)	93.6 (90.0, 97.2)	94.8 (92.4, 97.2)	93.8 (90.4, 97.2)	95.9 (94.1, 97.7)	94.9 (92.5, 97.3)
Experts	88.8 (85.6, 92.0)[0.005]	96.4 (93.0, 99.8)[0.298]	92.6 (90.8, 94.4)[0.193]	96.3 (93.0, 99.6)[0.346]	89.7 (87.2, 92.2)[0.004]	92.3 (90.4, 94.2)[0.132]
Emergency physicians	71.2 (66.0, 76.4)[0.003]	95.6 (91.8, 99.4)[0.889]	83.4 (80.0, 86.8)[0.005]	94.4 (89.8, 99.0)[0.768]	77.0 (73.5, 80.5)[0.002]	81.0 (76.9, 85.1)[0.005]

Numbers in parentheses are 95% CIs. Numbers in bracket are *p* values compared to AI model. PPV, positive predictive value; NPV, negative predictive value; AI, artificial intelligence; Cl, confidence interval.

**Table 4:** Diagnostic performance of AI on the external dataset.

External Dataset	Sensitivity (%)	Specificity (%)	Accuracy (%)	PPV (%)	NPV (%)	F1_Score (%)
2nd hospital	93.9 (91.6, 96.2)	94.2 (90.7, 97.7)	94.0 (92.0, 96.0)	93.9 (90.4, 97.4)	94.3 (92.3, 96.3)	93.8 (91.8, 95.8)

Numbers in parentheses are 95% CIs. CI, confidence interval; 2nd Hospital, The Second Hospital of Jilin University.

## Data Availability

The datasets generated and analyzed during the current study are not publicly available due to patient privacy and confidentiality restrictions under the ethical approval protocol. Anonymized data may be made available from the corresponding author upon reasonable request and with permission from the institutional ethics committee.
